# Promising results of revision total hip arthroplasty using a hexagonal, modular, tapered stem in cases of aseptic loosening

**DOI:** 10.1371/journal.pone.0233035

**Published:** 2020-06-23

**Authors:** Jan Schwarze, Christoph Theil, Georg Gosheger, Ralf Dieckmann, Burkhard Moellenbeck, Thomas Ackmann, Tom Schmidt-Braekling

**Affiliations:** 1 Department of Orthopedics and Tumor Orthopedics, Muenster University Hospital, Muenster, Albert-Schweitzer-Campus, Muenster, Germany; 2 Department of Orthopedics, Krankenhaus der Barmherzigen Brüder Trier, Nordallee, Germany; Seoul National University College of Medicine, REPUBLIC OF KOREA

## Abstract

**Background:**

Modular stems are widely used in revision total hip arthroplasty (THA) with aseptic loosening being a common reason for revision. Despite the good results reported on the use of modular stem designs, there are only few studies focusing on aseptic revisions and few studies on a hexagonal stem design. The goal of this study is to determine stem survival, clinical and functional outcome along with possible risk factors for implant failure in aseptic revision THA.

**Methods:**

We retrospectively identified 53 patients with aseptic THA revision using a modular hexagonal stem with a minimum follow-up of two years. Femoral bone loss, radiographic and clinical outcomes as well as function measured using the Harris Hip Score (HHS) was assessed. Patients’ previous medical history was analyzed for comorbidities and the body mass index. Stem survival was calculated using the Kaplan-Meier method. Possible risk factors for implant failure were analyzed using the log-rank test. The median age at revision was 69 (IQR 62–73) with a median follow up of 74 months.

**Results:**

Implant survival rates amounted to 90.4% at 3 and 5 years. The median HHS improved by 47 points (34 (IQR 22–47) vs 81 (IQR 59–90) p<0.001). There was a reduced implant survival after 5 years when the revision stem was used following a previous cemented stem (83.4% vs 100%, p = 0.04).

**Conclusion:**

A modular, hexagonal stem can be successfully used in aseptic revision THA with remarkable functional results and excellent survivorship. Revision of a cemented stem using this implant might result in reduced survival which must be considered when planning treatment.

## Introduction

Total hip arthroplasty (THA) is a successful treatment option for osteoarthritis and leads to excellent outcomes in the long-term [1]. However, there are several mechanisms of implant failure that might require revision surgery with approximately 50.000 THA revisions performed annually in the US [2]. By 2026 the number of THA revisions is expected to double making constant improvement indispensable to meet the growing demand in quality and quantity [3]. One of the most common reasons for revision surgery is aseptic loosening with 16.8% of revision THA performed for this indication. In 16.4% of all revisions in 2017 the stem was affected [4].

While there are several challenges in performing revision THA, femoral bone loss represents a major problem and complicates stable stem fixation [5–7]. While minor bone loss with intact metaphyseal region can be treated using conventional stems, major bone loss with deficient metaphyseal bone requires a different approach with several different cementless or cemented stems available to achieve stable fixation [8]. Bridging the bone defect using a cemented technique is possible however, higher loosening rates for cemented revision stems are discussed [9]. Alternatively, a diaphyseal anchorage using a cementless long stem can be used with multiple modular or non-modular, monoblock designs available. While one advantage of non-modular monobloc stems might be a reduced rate of intraoperative fractures, stem subsidence was found to be less common after implantation of a modular system [10]. However despite offering great intraoperative variability to address different defects and reconstruct the hip’s center of rotation as well as leg length irrespective of the distal fixation in cases of major bone loss [7,8,11,12], there are downsides to modularity with previous studies reporting potential mechanical failures of the junctional sites such as fretting, fatigue fractures or corrosion [11,13–15].

Current studies suggest a good mid to long-term survival rate between 82% and 96% after six [16] and ten years of follow-up [17] generally leading to a significant improvement of functional scores. The main causes of failure were recurrent periprosthetic joint infection (PJI) after septic revisions [17–19] and aseptic loosening [16].

However, previous studies report results of multiple stem designs used for heterogeneous septic and aseptic indications. The majority of the current studies describe stem survival and functional outcome of a modular system focusing on staged revision for PJI [17–27]. While aseptic loosening is a major cause for revision in THA, there is a scarcity of studies focusing on this indication. Furthermore, there is no study on using a hexagonal, modular stem design for aseptic indications to our knowledge.

The purpose of this study is to investigate survival rates of a modular revision stem in cases of aseptic revision total hip arthroplasty and identify potential risk factors for failure.

## Methods

Approval of the institutional review board (Ethik Kommission der Ärztekammer Westfalen-Lippe und der Westfälischen Wilhelmsuniversität) was obtained prior to this investigation (local ethical committee ref. no.2018-704-f-S). All data were fully anonymized before analysis. Patients medical records where accessed from December 2018 to April 2019 from our hospital database. A specific source of funding was not required in this study. It was conducted according to the principles of the World Medical Association Declaration of Helsinki.

**Fig 1:**
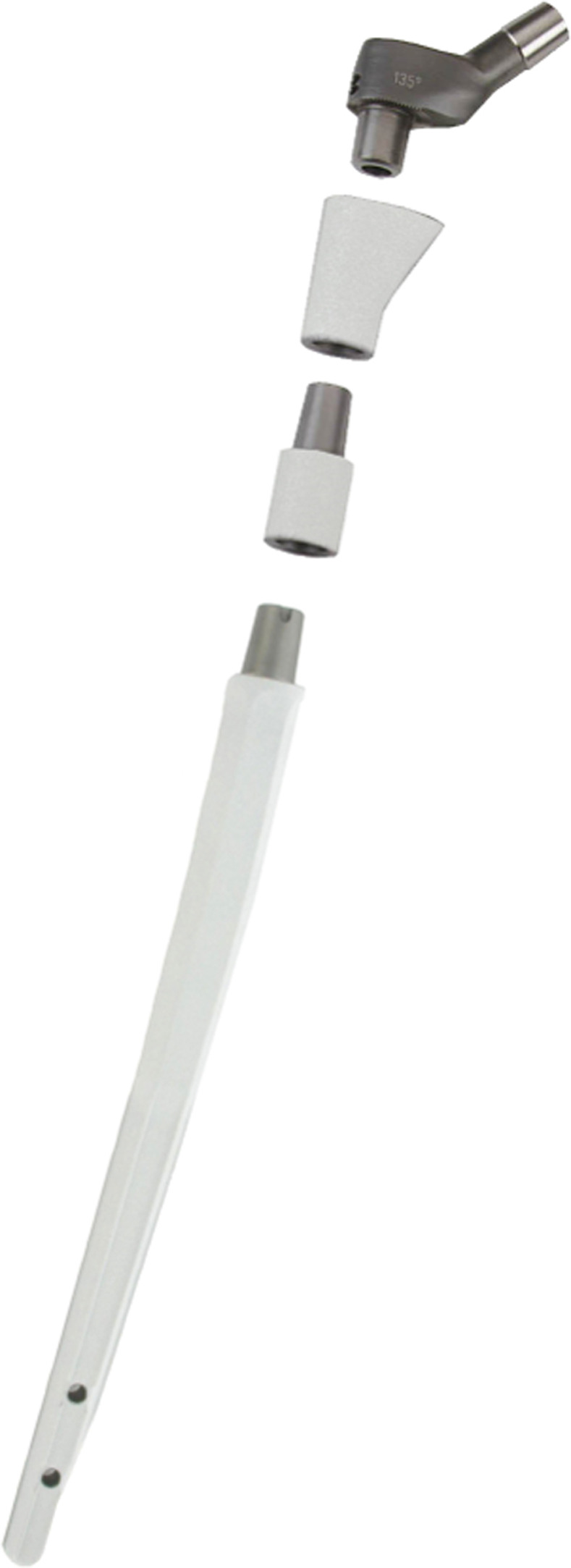
The modular tumor and revision system RS stem. This modular, hexagonal revision system has a hydroxyapatite (HA) coating on the metaphyseal and diaphyseal parts which allows a combined distal and proximal cementless fixation. Additionally, a non HA-coated version for a cemented fixation is available.

We retrospectively reviewed our institution’s database and identified all patients who underwent aseptic, single-stage revision THA using a single design hexagonal femoral revision stem (MUTARS–modular tumor and revision system RS stem, Implantcast GmbH, Buxtehude, Germany) (Fig 1) between 2006 and 2015.

Patients who were treated for periprosthetic joint infection, different stem designs or megaprosthetic reconstructions were excluded. A minimum follow-up period of 24 months was required. Follow-up was derived from the last contact with our institution.

Applying the exclusion criteria, we were able to identify 71 cases. 14 patients (19.7%) did not return to our clinic for their examination and four patients (8.5%) died during the first 24 month after exchange of the prosthesis of other cause to our knowledge. leaving 53 patients for further analysis. The median follow-up was 74 months (interquartile range (IQR) 31–90).

Patient’s previous medical history was analyzed for comorbidities and the body mass index (BMI) and Charlson Comorbidity Index (CCI) was calculated [28,29] (Table 1).

**Table 1:** Patients’ demographics.

Variable	Median	IQR^1^
**Age at surgery in years**	69	62–73
**Follow-up period in months**	72	38–91
**BMI in kg/m**^**2**^ *	29	24–35
**Charlson comorbidity index**	3	2–4

*Missing data in 3 cases, 1 IQR 25% to 75%

Preoperative conventional radiographs were obtained and femoral bone loss was assessed and classified using the Paprosky Index in cases of aseptic loosening [5,30,31]. In 5 cases of loosening combined with a periprosthetic fracture of the femur the Vancouver classification was used [32,33] (Table 2).

**Table 2:** Surgical details.

Variable	N	%
**Sex**		
**Male**	20	37.7
**Female**	33	62.3
**Indication for revision**		
**Aseptic loosening**	49	92.5
**Fracture**	4	7.5
**femoral bone loss [31]**		
**I**	2	3.8
**II**	15	28.3
**IIIa**	24	45.3
**IIIb**	6	11.3
**IV**	2	3.8
**Classification of fractures [33]**		
**B2**	1	1.9
**B3**	3	5.7
**Mode of fixation**		
**Cementless**	48	90.5
**Cemented**	5	9.5
**Concomitant cup revision**		
**Yes**	22	41.5
**No**	31	58.5

To rule out periprosthetic joint infection (PJI) preoperative joint aspiration and evaluation of serum C-reactive-protein (CRP), interleukin-6 [34] and leukocytes was performed [35]. In addition, intraoperative tissue samples were obtained and cultured for at least 7 to 14 days for microbiological growth.

**Fig 2:**
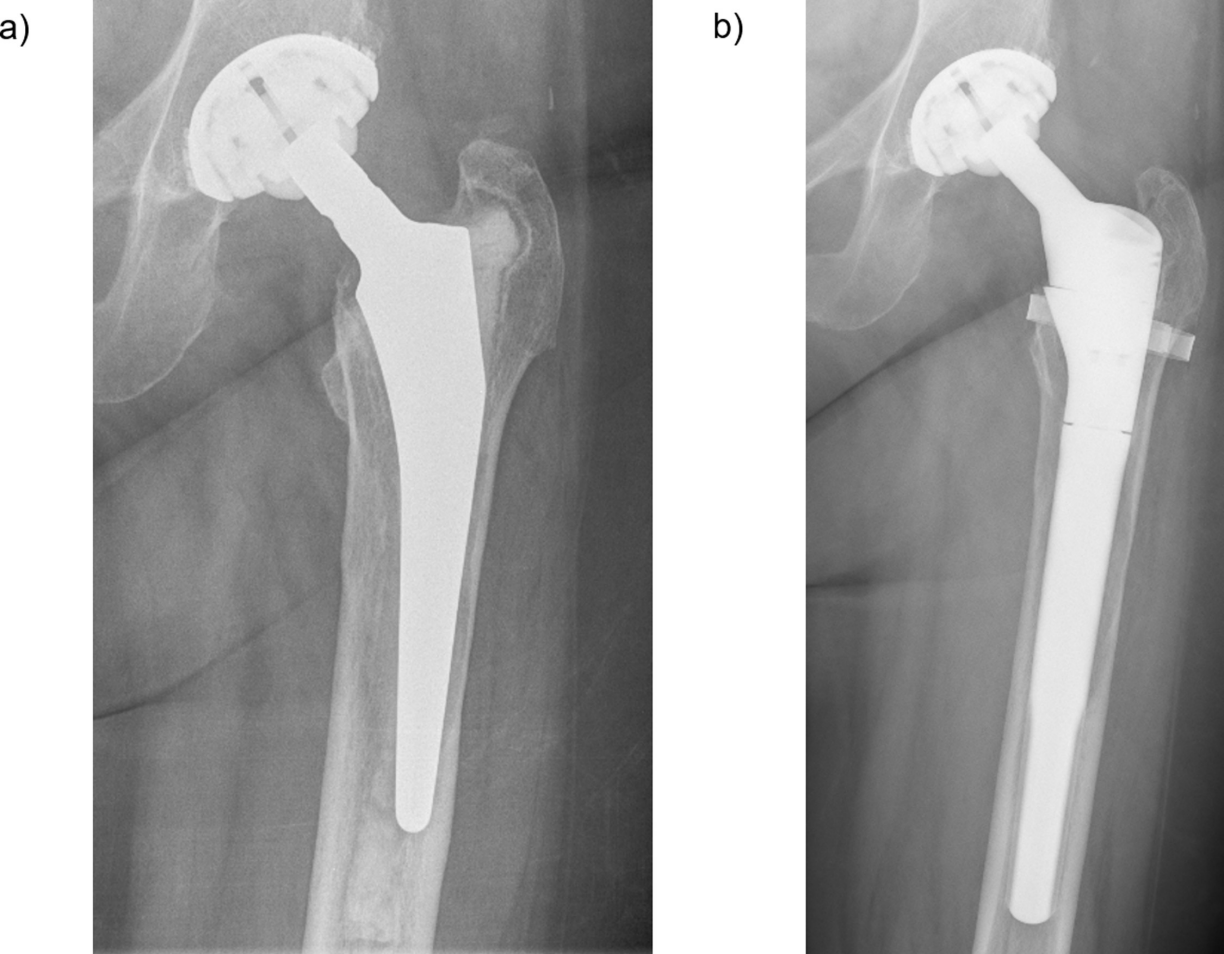
Pre- and postoperative radiographs. a) cemented primary THA with aseptic stem loosening and a Paprosky IIIa femoral bone defect due to the great amount of bone cement b) postoperative radiography with a uncemented MUTARS RS Stem.

All surgeries were performed by a senior orthopedic surgeon with expertise in revision arthroplasty. Synovectomy, debridement and pulsed irrigation were performed before implantation of the revision stem. In case of a loosening of a cemented stem, all remaining cement was completely removed. Postoperative all patients with uncemented stems were allowed partial weight bearing for six weeks. After a clinical examination and radiographic reevaluation, they were allowed to begin full weight bearing (Fig 2).

Primary endpoint was defined as prosthetic failure requiring revision of the stem component or exchange of the implant. Secondary endpoints were death and revision without implant removal.

Postoperative radiographs were examined for signs of loosening, subsidence and osteointegration using the criteria established by Engh et al. [36].

Functional assessment was performed using the Harris Hip Score (HHS) [37] preoperatively and at last clinical examination in our department for all retained stems.

### Statistical analysis

Data collection and statistical analysis were performed using Excel (Microsoft Corporation, Redmont, Washington, USA) and SPSS Statistics for Windows Version 25 (IBM Corporation, Armonk, NY, USA). All patient records were anonymized prior to analysis.

Data were analyzed according its distribution: means and ranges were calculated for parametric data; for nonparametric data medians and interquartile ranges (IQR) were calculated. Survival analysis was performed using the Kaplan-Meier method [38], differences in survival and influencing factors were assessed using the log-rank test [28]. Contingency tables were analyzed using the chi-squared-test. Differences in means were compared using the student’s t- test, non-parametric analyses were performed using the Mann-Whitney U-test and Wilcoxon signed rank test. Statistical significance was defined as p≤0.05.

## Results

### Implant survival and failure modes

**Fig 3:**
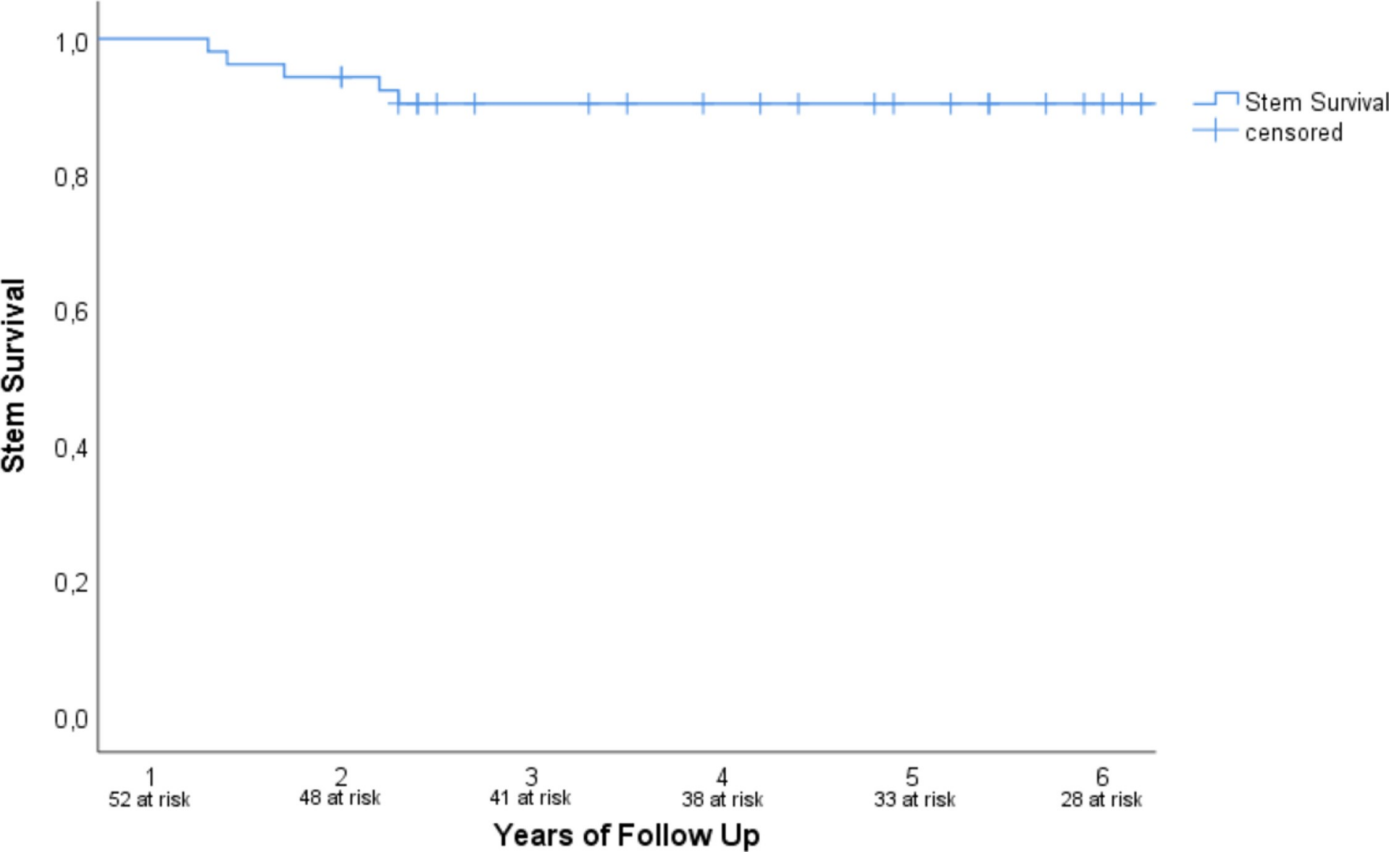
Stem survival after 6 years of follow up. Implant survival was found to be 90.4% (95% CI 82.4–98.4) at 3 and 5 years.

Implant survival was found to be 90.4% (95% CI 82.4–98.4%) at 3 and 5 years with 41 and 33 at risk. (Fig 3). Patients with a minimum follow up of 5 years displayed implant survival of 87.2% (95% CI 76.7–97.7%) after 5 years with 33 risk.

In three cases (5.7%) initial fixation failure of the stem with stem revision occurred. Loosening of the neck resulted in a revision of the prothesis without stem revision in one patient (1.9%) (Table 3).

**Table 3:** Patients with stem failure.

implant survival (month)	Reason	Paprosky	CCI	Revision implant
16	aseptic loosening	IIIb	2	total femur arthroplasty
17	neck-taper failure	IIIa	1	uncemented RS system^1^
21	aseptic loosening	IIIb	6	cemented monoblock
26	aseptic loosening	IV	2	cemented RS system
27	PJI	IIIb	4	cemented modular system*
147	PJI	II	3	cemented monoblock*

*outside hospital,

^1^ revision of the neck-taper junction without stem revision

Another patient presented with delayed wound healing with suspicion for early onset infection shortly after the operation and underwent surgical debridement, lavage and exchange of mobile components without stem revision. Six patients (11.3%) underwent revision of the cup because of aseptic loosening in our clinic during their follow up. The stem did not require revision in all six cases. While 11 cases (20.8%) showed intraoperative positive cultures, a PJI resulting in stem revision only occurred in two cases (3.8%) (after 27 and 147 months). Of the 11 patients with intraoperative positive cultures 5 received antibiotic treatment, while the rest were classified as contamination of the tissue sample.

**Fig 4:**
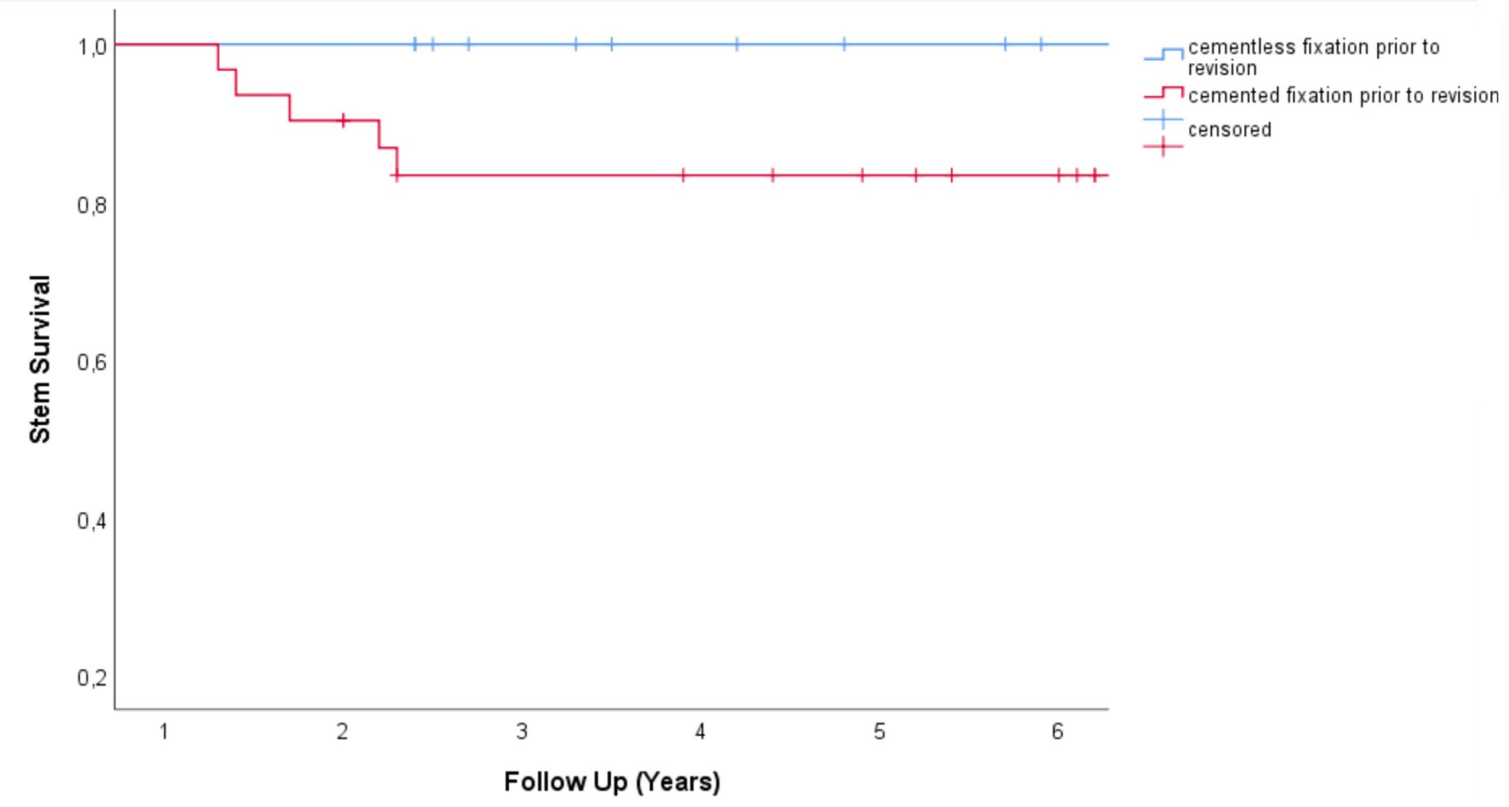
Stem survival compared regarding prior fixation of the revised stem. A cemented fixation of the stem in need for revisions turned out to be a significant risk factor for stem failure after 5 years of follow up of the MUTARS RS stem in our collective (100% (95% CI 100–100%) vs 83.4% (95% CI 70.1–96.7%) p = 0.04 log-rank test).

In our analysis of potential risk factors for stem revision we investigated obesity (BMI>30), age (≥70), sex, major bone loss (Paprosky ≥ IIIA), intraoperative positive cultures, and prior cementation of the femoral component. In all six cases of stem revision for any reason the previous stem was cemented (p = 0.04 Log-Rank-Test). With the numbers we had, we could not identify any further risk factors for stem revision. (Table 4 and Fig 4).

**Table 4:** Risk factor analysis.

Variable	N patients	5-year implant survival in %	95% CI	P (log-rank)
**Age**				0.54
**<70**	28	89.1	77.6–100	
**>70**	25	91.8	80.5–100	
**CCI > 5**				0.92
**yes**	10	90.0	71.4–100	
**no**	43	90.1	81.8–98.8	
**Obesity (BMI>30)**^1^				0.74
**yes**	20	90	76.9–100	
**no**	30	89.7	78.8–98.6	
**Prior stem fixation**				0.04
**cementless**	22	100	100–100	
**cemented**	31	83.4	70.1–96.7	
**Intraoperative positive cultures**				0.92
**yes**	11	90.9	73.9–100	
**no**	42	90.3	73.9–100	
**Major Bone Loss (Paprosky ≥ IIIa)**				0.24
**yes**	36	85.8	74.3–97.3	
**no**	17	100	100–100	

^1^three missing values for BMI

### Functional outcome

The Harris Hip Score (HHS) was available in 40 cases. The median HHS improved by 47 points after stem revision (34 (IQR 22–47) vs. 81 (IQR 59–90) points; nonparametric distribution, p<0.001 Wilcoxon signed rank test). No significant difference in HSS were observed between cohorts based on bone loss, cementation, stem length or diameter, comorbidities, obesity, sex or age in our analysis (Mann Whitney U-Test).

## Discussion

This is a retrospective, single center, single prosthetic design study investigating implant survival and functional outcome of a modular revision stem. The main finding was that a single design hexagonal stem shows excellent mid and long-term survival and improvement of function. While potential patient related risk factors could not be identified, cementation of the previously implanted stem before revision surgery was associated with a significant shorter revision free survival of the RS stem.

In a constantly aging society mobility of the elderly remains a topic of growing popularity. To meet these expectations THA revision surgery will have to undergo constant improvement. Bone loss of the proximal femur is one of the greatest challenges regarding stem fixation [5,30]. Modular tapered stems for a cementless distal fixation are the preferred method for hip revision surgery in cases of major bone loss [7,22].

While this is a single-center, study using a single design modular implant only for aseptic cases with a good mid- to long-term follow-up, there are several limitations to this study: It is limited by its retrospective design with possible recall and selection bias and small numbers in the group of cemented stems making comparison of these two fixation methods difficult with the numbers available. Furthermore, given that there were only few failures we decided not to do a multivariate analysis to potentially confirm the results found in univariate analysis.

We found a 90.4% survivorship free from stem revision after 3 and 5 years which is comparable to other studies that report 82–96% mid to long-term survival [17,16,19,21,26]. Concurring with a previous study, we were not able to identify patient-related factors such as age, sex, and obesity as significant for implant survival [16]. Whereas a prior cementation of the stem turned out to be a risk factor for LOS of the revision (p = 0.04) in our collective. To our knowledge, there is no study that has discussed this association. While other study does not allow a more detailed identification of the reasons for this finding, 4 out of 6 of these patients had type IIIb or worse bone loss that needed to be addressed. This might be due to the fact that previous cementation was chosen because of poor bone quality or to reconstruct an existing defect in the previous surgery. Furthermore, the complete removal of the previous cementation might have caused additional damage to the host bone. In these cases, additional procedures such as impaction bone grafting [39,40] or repeat cementation in an existing residually cemented medullary canal might be an option [41,42].

While modular systems offer a variety of surgical and biomechanical advantages [7,8,11], modular junctions bear the risk of mechanical failure due to corrosion and fretting [11,13–15]. In our collective one case of mechanical failure regarding the stem-taper junction occurred and had to be revised. The implant did not show any signs of corrosion. Retrospectively the fixation failure could most likely be linked to a loosening of the connective screw possibly due to deficient assembly of the parts. No case of stem or neck-fracture occurred. Nevertheless patients certainly need to be counseled regarding this mode of failure particularly when other risk factors such as obesity and lack of proximal bone support leading to increased bending forces are present [43,44].

The functional outcome in our collective measured by a median HHS of 81 points at last follow-up and significant improvement of 47 points (p<0.001) shows a comparable result to previous studies [26]. Postoperative Harris Hip Scores show a similar distribution between 72 and 76 with an improvement from 25 to 37 points in the literature following revision with a modular fluted tapered stem [16,18,19,26].

PJI after revision surgery is one of the main causes for implant failure, increases the risk for further revisions and subsequent complications [21,45]. Despite the fact that every patient underwent a preoperative diagnostic algorithm to identify a possible infection, we found a relevant number of patients with unsuspected positive cultures (20.8%) in intraoperative samples. While with the the numbers we had, intraoperative positive cultures were not associated with a reduced implant survival which is in line with a previous study [46], the role of unsuspected positive cultures in revision surgery of THA and TKA remains a topic of interest to further studies. We recommend optimization of potential risk factors for PJI in every revision patient such as BMI, diabetes and skin decolonisation and conduct a thorugh preoperative diagnostic algorithm to rule out infection although this might not be universally possible [47].

## Conclusion

A modular, hexagonal revision system can be successfully used in revision THA with very good functional results and excellent survivorship even in cases of major bone defects of the proximal femur. While previous cementation might be a risk factor for stem revision, failure of the modular components was rare, nonetheless both factors among others must be considered in planning and should be discussed with patients.

## Supporting information

S1 File Full data set for manuscript. (PDF)

## Abbreviations

BMI: body mass index in kg/m²
CCI: Charlson Comorbidity Index
CI: confidence interval
CRP: C reactive protein
HA: hydroxylapatite
HHS: Harris Hip Score
IQR: interquartile range 25% - 75%
MUTARS: modular universal tumor and revision system
PJI: periprosthetic joint infection
THA: total hip arthroplasty

## References

[1] Mariconda M, Galasso O, Costa GG, Recano P, Cerbasi S. Quality of life and functionality after total hip arthroplasty: a long-term follow-up study. BMC Musculoskelet Disord 2011;12:1–10. 10.1186/1471-2474-12-1 21978244 PMC3204273

[2] Gwam CU, Mistry JB, Mohamed NS, Thomas M, Bigart KC, Mont MA et al Current Epidemiology of Revision Total Hip Arthroplasty in the United States: National Inpatient Sample 2009 to 2013. The Journal of Arthroplasty 2017;32:2088–92. 10.1016/j.arth.2017.02.046 28336249

[3] Kurtz S, Ong K, Lau E, Mowat F, Halpern M. Projections of primary and revision hip and knee arthroplasty in the United States from 2005 to 2030. The Journal of bone and joint surgery. American volume 2007;89:780–5. 10.2106/JBJS.F.00222 17403800

[4] Grimberg Alexander, Jansson Volkmar, Liebs Thoralf, Melsheimer Oliver, Arnd Steinbrück. Endoprothesenregister Deutschland (EPRD) Jahresbericht 2017.

[5] Sheth NP, Nelson CL, Paprosky WG. Femoral bone loss in revision total hip arthroplasty: evaluation and management. The Journal of the American Academy of Orthopaedic Surgeons 2013;21:601–12. 10.5435/JAAOS-21-10-601 24084434

[6] Sculco PK, Abdel MP, Lewallen DG. Management of femoral bone loss in revision total hip arthroplasty. Hip international the journal of clinical and experimental research on hip pathology and therapy 2015;25:380–7. 10.5301/hipint.5000272 26351121

[7] Sakellariou VI, Babis GC. Management bone loss of the proximal femur in revision hip arthroplasty: Update on reconstructive options. World journal of orthopedics 2014;5:614–22. 10.5312/wjo.v5.i5.614 25405090 PMC4133469

[8] Gravius S, Randau T, Wirtz DC. What can be done when hip prostheses fail? New trends in revision endoprosthetics. Der Orthopade 2011;40:1084–94. 10.1007/s00132-011-1844-0 22109589

[9] Wirtz DC, Niethard FU. Etiology, diagnosis and therapy of aseptic hip prosthesis loosening—a status assessment. Zeitschrift fur Orthopadie und ihre Grenzgebiete 1997;135:270–80. 10.1055/s-2008-1039388 9381761

[10] Huang Y, Zhou Y, Shao H, Gu J, Tang H, Tang Q. What Is the Difference Between Modular and Nonmodular Tapered Fluted Titanium Stems in Revision Total Hip Arthroplasty. The Journal of Arthroplasty 2017;32:3108–13. 10.1016/j.arth.2017.05.021 28602532

[11] Srinivasan A, Jung E, Levine BR. Modularity of the femoral component in total hip arthroplasty. The Journal of the American Academy of Orthopaedic Surgeons 2012;20:214–22. 10.5435/JAAOS-20-04-214 22474091

[12] Gosheger G, Gebert C, Ahrens H, Streitbuerger A, Winkelmann W, Hardes J. Endoprosthetic reconstruction in 250 patients with sarcoma. Clinical orthopaedics and related research 2006;450:164–71. 10.1097/01.blo.0000223978.36831.39 16691142

[13] Nawabi DH, Do HT, Ruel A, Lurie B, Elpers ME, Wright T et al Comprehensive Analysis of a Recalled Modular Total Hip System and Recommendations for Management. The Journal of bone and joint surgery. American volume 2016;98:40–7. 10.2106/JBJS.N.01121 26738902

[14] Konan S, Garbuz DS, Masri BA, Duncan CP. Modular tapered titanium stems in revision arthroplasty of the hip: The Risk and Causes of Stem Fracture. The bone & joint journal 2016;98-B:50–3. 10.1302/0301-620X.98B1.36442 26733641

[15] Su SL, Koch CN, Nguyen TM, Burket JC, Wright TM, Westrich GH. Retrieval Analysis of Neck-Stem Coupling in Modular Hip Prostheses. The Journal of Arthroplasty 2017;32:2301–6. 10.1016/j.arth.2017.02.016 28262451

[16] Abdel MP, Cottino U, Larson DR, Hanssen AD, Lewallen DG, Berry DJ. Modular Fluted Tapered Stems in Aseptic Revision Total Hip Arthroplasty. The Journal of bone and joint surgery. American volume 2017;99:873–81. 10.2106/JBJS.16.00423 28509828

[17] Smith MA, Deakin AH, Allen D, Baines J. Midterm Outcomes of Revision Total Hip Arthroplasty Using a Modular Revision Hip System. The Journal of Arthroplasty 2016;31:446–50. 10.1016/j.arth.2015.08.029 26432674

[18] Hoberg M, Konrads C, Engelien J, Oschmann D, Holder M, Walcher M et al Outcome of a modular tapered uncemented titanium femoral stem in revision hip arthroplasty. International orthopaedics 2015;39:1709–13. 10.1007/s00264-015-2699-5 25690924

[19] Jibodh SR, Schwarzkopf R, Anthony SG, Malchau H, Dempsey KE, Estok DM. Revision hip arthroplasty with a modular cementless stem: mid-term follow up. The Journal of Arthroplasty 2013;28:1167–72. 10.1016/j.arth.2012.07.031 23114191

[20] Houdek MT, Perry KI, Wyles CC, Berry DJ, Sierra RJ, Trousdale RT. Use of a Modular Tapered Fluted Femoral Component in Revision Total Hip Arthroplasty Following Resection of a Previously Infected Total Hip: Minimum 5-year Follow-Up. The Journal of Arthroplasty 2015;30:435–8. 10.1016/j.arth.2014.08.012 25534955

[21] Dieckmann R, Schulz D, Gosheger G, Becker K, Daniilidis K, Streitbürger A et al Two-stage hip revision arthroplasty with a hexagonal modular cementless stem in cases of periprosthetic infection. BMC musculoskeletal disorders 2014;15:398 10.1186/1471-2474-15-398 25428415 PMC4289174

[22] Dzaja I, Lyons MC, McCalden RW, Naudie DDD, Howard JL. Revision Hip Arthroplasty Using a Modular Revision Hip System in Cases of Severe Bone Loss. The Journal of Arthroplasty 2014;29:1594–7. 10.1016/j.arth.2014.02.035 24768500

[23] Krishnamurthy AB, MacDonald SJ, Paprosky WG. 5- to 13-year follow-up study on cementless femoral components in revision surgery. The Journal of Arthroplasty 1997;12:839–47. 10.1016/s0883-5403(97)90152-2 9458248

[24] Moreta J, Uriarte I, Foruria X, Loroño A, Agirre U, Jáuregui I et al Medium-term outcomes of the S-ROM modular femoral stem in revision hip replacement. European journal of orthopaedic surgery & traumatology orthopedie traumatologie 2018;28:1327–34. 29696415 10.1007/s00590-018-2213-z

[25] Restrepo C, Mashadi M, Parvizi J, Austin MS, Hozack WJ. Modular femoral stems for revision total hip arthroplasty. Clinical orthopaedics and related research 2011;469:476–82. 10.1007/s11999-010-1561-8 20865464 PMC3018211

[26] Lakstein D, Kosashvili Y, Backstein D, Safir O, Lee P, Gross AE. Revision total hip arthroplasty with a modular tapered stem. HIP International 2010;20:136–42. 10.1177/112070001002000202 20544661

[27] Rieger B, Ilchmann T, Bolliger L, Stoffel KK, Zwicky L, Clauss M. Mid-term results of revision total hip arthroplasty with an uncemented modular femoral component. Hip international the journal of clinical and experimental research on hip pathology and therapy 2018;28:84–9. 10.5301/hipint.5000522 PMC6159842 29027190

[28] Charlson ME, Pompei P, Ales KL, MacKenzie CR. A new method of classifying prognostic comorbidity in longitudinal studies: development and validation. Journal of chronic diseases 1987;40:373–83. 10.1016/0021-9681(87)90171-8 3558716

[29] Quan H, Li B, Couris CM, Fushimi K, Graham P, Hider P et al Updating and validating the Charlson comorbidity index and score for risk adjustment in hospital discharge abstracts using data from 6 countries. American journal of epidemiology 2011;173:676–82. 10.1093/aje/kwq433 21330339

[30] Ibrahim DA, Fernando ND. Classifications In Brief: The Paprosky Classification of Femoral Bone Loss. Clinical orthopaedics and related research 2017;475:917–21. 10.1007/s11999-016-5012-z 27484412 PMC5289194

[31] Aribindi R, Barba M, Solomon MI, Arp P, Paprosky W. Bypass fixation. The Orthopedic clinics of North America 1998;29:319–29. 10.1016/s0030-5898(05)70330-8 9553577

[32] Gaski GE, Scully SP. In brief: classifications in brief: Vancouver classification of postoperative periprosthetic femur fractures. Clinical orthopaedics and related research 2011;469:1507–10. 10.1007/s11999-010-1532-0 20809166 PMC3069264

[33] Duncan CP, Masri BA. Fractures of the femur after hip replacement. Instructional course lectures 1995;44:293–304. 7797866

[34] Hoell S, Borgers L, Gosheger G, Dieckmann R, Schulz D, Gerss J et al Interleukin-6 in two-stage revision arthroplasty: what is the threshold value to exclude persistent infection before re-implanatation? The bone & joint journal 2015;97-B:71–5. 25568416 10.1302/0301-620X.97B1.33802

[35] Parvizi J, Suh D-H, Jafari SM, Mullan A, Purtill JJ. Aseptic Loosening of Total Hip Arthroplasty: Infection Always Should be Ruled Out. Clinical orthopaedics and related research 2011;469:1401–5. 10.1007/s11999-011-1822-1 21365337 PMC3069261

[36] Engh CA, Bobyn JD, Glassman AH. Porous-coated hip replacement. The factors governing bone ingrowth, stress shielding, and clinical results. The Journal of bone and joint surgery. British volume 1987;69:45–55. 3818732 10.1302/0301-620X.69B1.3818732

[37] Harris WH. Traumatic arthritis of the hip after dislocation and acetabular fractures: treatment by mold arthroplasty. An end-result study using a new method of result evaluation. The Journal of bone and joint surgery. American volume 1969;51:737–55. 5783851

[38] Kaplan EL, Meier P. Nonparametric Estimation from Incomplete Observations. Journal of the American Statistical Association 1958;53:457.

[39] Oakes DA, Cabanela ME. Impaction bone grafting for revision hip arthroplasty: biology and clinical applications. The Journal of the American Academy of Orthopaedic Surgeons 2006;14:620–8. 10.5435/00124635-200610000-00004 17030595

[40] Wimmer MD, Randau TM, Deml MC, Ascherl R, Nöth U, Forst R et al Impaction grafting in the femur in cementless modular revision total hip arthroplasty: a descriptive outcome analysis of 243 cases with the MRP-TITAN revision implant. BMC musculoskeletal disorders 2013;14:19 10.1186/1471-2474-14-19 23311769 PMC3556053

[41] Sandiford NA, Jameson SS, Wilson MJ, Hubble MJW, Timperley AJ, Howell JR. Cement-in-cement femoral component revision in the multiply revised total hip arthroplasty: results with a minimum follow-up of five years. The bone & joint journal 2017;99-B:199–203. 28148661 10.1302/0301-620X.99B2.BJJ-2016-0076.R1

[42] Cnudde PHJ, Kärrholm J, Rolfson O, Timperley AJ, Mohaddes M. Cement-in-cement revision of the femoral stem: analysis of 1179 first-time revisions in the Swedish Hip Arthroplasty Register. The bone & joint journal 2017;99-B:27–32. 28363891 10.1302/0301-620X.99B4.BJJ-2016-1222.R1

[43] Lakstein D, Eliaz N, Levi O, Backstein D, Kosashvili Y, Safir O et al Fracture of cementless femoral stems at the mid-stem junction in modular revision hip arthroplasty systems. The Journal of bone and joint surgery. American volume 2011;93:57–65. 10.2106/JBJS.I.01589 21209269

[44] Fink B. What can the surgeon do to reduce the risk of junction breakage in modular revision stems? Arthroplasty today 2018;4:306–9. 10.1016/j.artd.2018.03.002 30186910 PMC6123316

[45] Bozic KJ, Ries MD. The impact of infection after total hip arthroplasty on hospital and surgeon resource utilization. The Journal of bone and joint surgery. American volume 2005;87:1746–51. 10.2106/JBJS.D.02937 16085614

[46] Jacobs AME, Bénard M, Meis JF, van Hellemondt G, Goosen JHM. The unsuspected prosthetic joint infection incidence and consequences of positive intra-operative cultures in presumed aseptic knee and hip revisions. The bone & joint journal 2017;99-B:1482–9. 29092987 10.1302/0301-620X.99B11.BJJ-2016-0655.R2

[47] Parvizi J, Tan TL, Goswami K, Higuera C, Della Valle C, Chen AF et al The 2018 Definition of Periprosthetic Hip and Knee Infection: An Evidence-Based and Validated Criteria. The Journal of Arthroplasty 2018;33:1309-1314.e2. 10.1016/j.arth.2018.02.078 29551303

