# Supplementary material for: Promising results of revision total hip arthroplasty using a hexagonal, modular, tapered stem in cases of aseptic loosening
Source: PLoS One. 2020 Jun 23;15(6):e0233035. doi: 10.1371/journal.pone.0233035 (PMC7310743; doi:10.1371/journal.pone.0233035)
Supplement: S1 File — (PDF) [file pone.0233035.s001.pdf]

Age	Follow up (months)	CCI	Paprosky / Va	prior cement	cemented	Harris Pre OP
64	147	3	II	1	0	
71	139	2	IIIa	1	0	34
63	80	3	IIIa	1	0	22
70	148	3	II	0	0	46
53	147	2	IIIa	1	0	21
75	21	6	IIIb	1	0	
73	78	3	IIIa	1	0	7
68	71	2	IIIa	0	0	
71	137	3	II	0	0	46
52	137	1	IIIa	0	0	53
66	136	2	IIIa	0	0	37
69	134	5	IIIa	0	0	59
53	28	2	IIIa	0	0	
69	39	2	IIIa	0	0	97
68	124	3	IIIa	0	0	35
60	113	1	IIIa	1	0	46
61	107	3	II	0	0	35
79	99	3	IIIa	1	0	29
71	85	3	II	1	0	32
62	30	3	I	0	0	
73	83	2	IIIa	0	0	35
71	80	3	II	1	0	32
62	26	2	IV	1	0	
67	94	2	II	1	0	24
70	74	3	IIIa	1	0	30
50	85	5	II	0	0	39
75	90	4	II	1	0	24
72	24	3	IIIa	1	0	
71	29	11	B3	0	0	
60	83	1	II	0	0	63
76	46	5	II	1	0	
63	82	2	IIIb	1	0	19
51	80	1	II	0	0	0
62	79	5	IIIa	1	0	25
69	73	2	II	1	0	35
72	72	4	IIIa	1	0	35
65	16	2	IIIb	1	0	
82	64	7	IIIb	1	0	19
77	51	3	B3	0	1	0
77	62	6	I	1	0	19
60	69	2	IIIb	0	0	30
78	64	3	IIIa	1	0	
67	74	2	IIIa	1	0	48
74	32	3	II	0	0	
77	59	3	IIIa	1	0	25
72	57	4	II	0	0	52
56	17	1	IIIa	1	0	50
89	53	5	B3	1	1	0
61	24	7	IIIa	1	0	22

68	42	3	B2	0	1	0
71	27	4	IIIb	1	0	
80	27	4	IIIa	1	0	50
63	89	2	IV	0	1	35

Harris Post OP	BMI	loss of implant
	27,4	1
31	23,9	0
32	22,7	0
81	33,5	0
84	29,4	0
	29,1	1
44	28,2	0
	27,3	0
81	33,5	0
97	31,6	0
75	34,7	0
86	32,7	0
	26,0	0
94	20,0	0
100		0
93	22,0	0
65	32,6	0
81	22,7	0
85	32,9	0
	26,3	0
82	24,5	0
85	32,9	0
	27,18	1
99	33,81	0
61	28,70	0
99	30,60	0
34	22,23	0
	28,73	0
	26,99	0
90	44,98	0
	25,71	0
89	28,96	0
56	42,56	0
25	30,09	0
91		0
68	31,21	0
	19,11	1
48	36,05	0
57	28,60	0
47	39,30	0
66	19,10	0
	n.e.	0
88	21,48	0
	21,83	0
84	23,66	0
67	30,37	0
92	30,12	1
69	29,04	0
68	26,87	0

53	21,19	0
	30,93	1
77	32,32	0
82	27,18	0
